# The Impact of Human Immunodeficiency Virus and Menopause on Bone Mineral Density: A Longitudinal Study of Urban‐Dwelling South African Women

**DOI:** 10.1002/jbmr.4765

**Published:** 2023-02-01

**Authors:** Tafadzwa Madanhire, Julia H. Goedecke, Kate A. Ward, Nicole Jaff, Nigel J. Crowther, Shane Norris, Rashida A. Ferrand, Andrea M. Rehman, Lisa K. Micklesfield, Celia L. Gregson

**Affiliations:** ^1^ SAMRC/Wits Developmental Pathways for Health Research Unit, School of Clinical Medicine, Faculty of Health Sciences University of the Witwatersrand Johannesburg South Africa; ^2^ Biomedical Research and Training Institute Harare Zimbabwe; ^3^ MRC International Statistics and Epidemiology Group, Department of Infectious Disease Epidemiology, Faculty of Epidemiology and Population Health London School of Hygiene and Tropical Medicine London UK; ^4^ Non‐Communicable Diseases Research Unit South African Medical Research Council Cape Town South Africa; ^5^ MRC Lifecourse Epidemiology Centre, School of Human Development and Health University of Southampton Southampton UK; ^6^ Department of Chemical Pathology, Faculty of Health Sciences University of the Witwatersrand Johannesburg South Africa; ^7^ Department of Chemical Pathology National Health Laboratory Service Johannesburg South Africa; ^8^ Global Health Research Institute, School of Human Development and Health University of Southampton Southampton UK; ^9^ Clinical Research Department, Faculty of Infectious and Tropical Diseases London School of Hygiene and Tropical Medicine London UK; ^10^ Musculoskeletal Research Unit, Translational Health Sciences, Bristol Medical School University of Bristol Bristol UK

**Keywords:** HUMAN IMMUNODEFICIENCY VIRUS, BONE MINERAL DENSITY, DUAL‐ENERGY X‐RAY ABSORPTIOMETRY, MENOPAUSE, SOUTH AFRICA

## Abstract

An estimated 25% of South African women live with human immunodeficiency virus (HIV). Antiretroviral therapy roll‐out has improved life expectancy, so many more women now reach menopause. We aimed to quantify changes in bone mineral density (BMD) during the menopausal transition in urban‐dwelling South African women with and without HIV and determine whether HIV infection modified the effect of menopause on BMD changes. A 5‐year population‐based longitudinal study recruited women aged 40–60 years residing in Soweto and collected demographic and clinical data, including HIV status, anthropometry, and BMD, at baseline and at 5‐year follow‐up. All women were staged as pre‐, peri‐, or postmenopausal at both time points. Multivariable linear regression assessed relationships and interactions between HIV infection, menopause, and change in BMD. At baseline, 450 women had mean age 49.5 (SD 5.7) years, 65 (14.4%) had HIV, and 140 (31.1%), 119 (26.4%), and 191 (42.4%) were pre‐, peri‐, and postmenopausal, respectively; 34/205 (13.6%) women ≥50 years had a total hip (TH) or lumbar spine (LS) *T*‐score ≤ −2.5. At follow‐up 38 (8.4%), 84 (18.7%), and 328 (72.9%) were pre‐, peri‐, and postmenopausal. Those with HIV at baseline lost more total body (TB) BMD (mean difference −0.013 [95% confidence interval −0.026, −0.001] g/cm^2^, *p* = 0.040) and gained more weight 1.96 [0.32, 3.60] kg; *p* = 0.019 than HIV‐uninfected women. After adjusting for age, baseline weight, weight change, and follow‐up time, the transition from pre‐ to postmenopause was associated with greater TB BMD losses in women with HIV (−0.092 [−0.042, −0.142] g/cm^2^; *p* = 0.001) than without HIV (−0.038 [−0.016, −0.060] g/cm^2^, *p* = 0.001; interaction *p* = 0.034). Similarly, in women who were postmenopausal at both time points, those with HIV lost more TB BMD (−0.070 [−0.031, −0.108], *p* = 0.001) than women without HIV (−0.036 [−0.015, −0.057], *p* = 0.001, interaction *p* = 0.049). Findings were consistent but weaker at the LS and TH. Menopause‐related bone loss is greater in women with HIV, suggesting women with HIV may be at greater risk of osteoporotic fractures. HIV services should consider routine bone health assessment in midlife women as part of long‐term HIV care delivery. © 2023 The Authors. *Journal of Bone and Mineral Research* published by Wiley Periodicals LLC on behalf of American Society for Bone and Mineral Research (ASBMR).

## Introduction

Of the nearly 38 million people living with human immunodeficiency virus (HIV) globally, two thirds live in sub‐Saharan Africa (SSA).^(^ ^1^ ^)^ Successful antiretroviral therapy (ART) roll‐out for people with HIV has dramatically improved survival,^(^ ^2^ ^)^ and life expectancy is increasing more rapidly across SSA than any other region.^(^ ^3^ ^)^ Taken together, all this means that there is an aging cohort of individuals with HIV at risk of chronic comorbidities.^(^ ^4^ ^)^ Although infectious diseases still account for the majority of deaths in midlife black women, SSA is experiencing an epidemiological transition with an increase in mortality rates attributable to noncommunicable disease.^(^ ^5^ ^)^ Furthermore, HIV itself may increase the risk of some chronic comorbidities, but population‐based data illustrating this are sparse in SSA. Such data are required to inform coordinated, long‐term integrated care management.^(^ ^6^ ^)^

Osteoporosis, a chronic disease of aging, is increasingly evident in older African populations, manifesting in fragility fractures of the spine and hip,^(^ ^7^, ^8^ ^)^ the latter being associated with high mortality in black South Africans.^(^ ^9^ ^)^ While many osteoporosis risk factors are similar across populations, including increasing age, menopause, low body mass index (BMI), and physical inactivity,^(^ ^10^, ^11^ ^)^ others may be more important in SSA.^(^ ^12^ ^)^ For example, HIV infection affects bone through a variety of mechanisms: directly through dysregulated systemic immune activation increasing bone turnover,^(^ ^13^ ^)^ indirectly through associated malnutrition, social deprivation, and/or opportunistic infection(s),^(^ ^14^ ^)^ and through HIV treatment itself.^(^ ^15^ ^)^ In South Africa, ART drugs such as tenofovir disoproxil fumarate (TDF) (part of first‐line regimens) may promote bone loss at the hip and spine.^(^ ^16^, ^17^, ^18^, ^19^ ^)^ A small study of Hispanic and African American women suggested that postmenopausal bone loss may be greater in women living with HIV, particularly in those with low BMI.^(^ ^20^ ^)^ It remains to be determined whether the same holds true in southern Africa, where the prevalence of both HIV and female overweight and obesity is high.^(^ ^21^, ^22^ ^)^

We hypothesized that HIV infection increases bone mineral density (BMD) loss as women transition through menopause. Hence, we first aimed to quantify changes in BMD, in urban‐dwelling South African women living with and without HIV as they transition through menopause. Second, we aimed to determine whether HIV infection modified the effect of menopause on BMD losses.

## Materials and Methods

### Study population

**Fig. 1:**
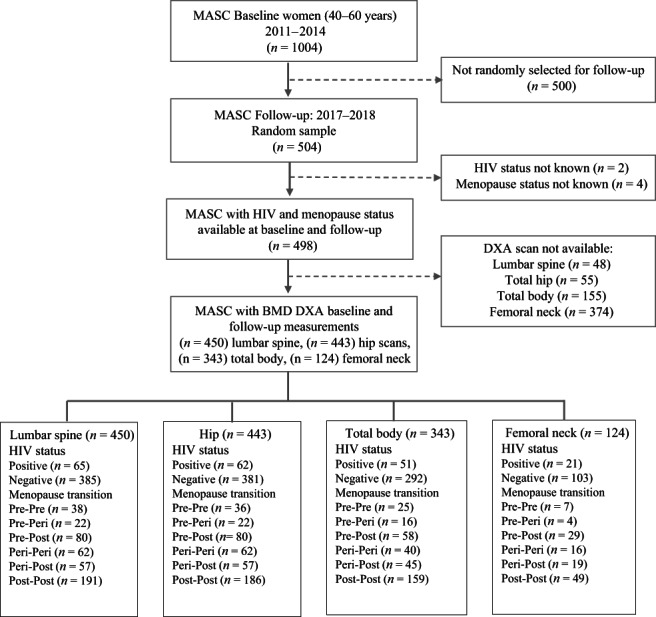
Participant recruitment flow diagram explaining selection of women in Middle‐Aged Soweto Cohort (MASC) study. The flow chart shows the selection of participants in the MASC study at baseline and follow‐up. In addition, the figure summarizes the distribution of data by BMD site, menopause group, and HIV status.

This longitudinal study was conducted at the South African Medical Research Council (SAMRC)/Wits Developmental Pathways for Health Research Unit, based at the Chris Hani Baragwanath Hospital in Soweto, Johannesburg, South Africa (SA). Baseline data collection took place between 2011 and 2015 as part of the AWI‐Gen (Africa Wits‐INDEPTH partnerships for Genomic Research) study,^(^ ^23^, ^24^ ^)^ which had randomly recruited 1004 caregivers of the Birth to Twenty Plus cohort.^(^ ^25^ ^)^ This cohort was followed up (January 2017 to August 2018) to study type 2 diabetes and included a randomly selected subsample of 510 women, then referred to as the Middle‐Aged Soweto Cohort (MASC).^(^ ^26^ ^)^ Dual energy X‐ray absorptiometry (DXA) scans were performed to determine body composition and BMD; the current study constitutes a secondary analysis. We included women aged 40–60 years at baseline, with available baseline and follow‐up data, which included assessment of menopausal status, DXA measurement of BMD, and known HIV status (Figure 1).

### Data collection

An interviewer‐administered questionnaire collected data on sociodemographic characteristics and medical history at both baseline and follow‐up using REDCap electronic data capture tools hosted at the University of the Witwatersrand.^(^ ^24^, ^27^, ^28^ ^)^ Anthropometry, grip strength, and DXA measurements were performed, together with HIV testing, as described in what follows.

### Menopausal status

Participants were classified at baseline and follow‐up into one of three menopause categories based on self‐reported final menstrual period (FMP).^(^ ^29^, ^30^ ^)^ Women currently having regular periods were classified as premenopausal, women having irregular periods were classified as perimenopausal, and women who had had no bleeding for more than 12 months were classified as postmenopausal. Hysterectomy prevents menopausal staging using menstrual bleeding criteria.^(^ ^30^, ^31^ ^)^ Estrogen is a hormone with both anabolic and antiresorptive effects on bone,^(^ ^32^ ^)^ and following menopausal transition estrogen production declines, while following oophorectomy it abruptly ceases.^(^ ^33^ ^)^ Therefore, the few women who reported a hysterectomy +/− oophorectomy within the last 12 months were added to the perimenopausal group (*n* = 1) (as their skeleton had experienced only 1 year or less of estrogen loss), and if they reported a hysterectomy more than a year ago, they were added to the postmenopausal group (*n* = 37), with preplanned sensitivity analyses (see subsequent discussion). Six menopause transition (MT) categories were generated according to the participant's baseline and follow‐up menopause grouping (pre‐pre, pre‐peri, pre‐post, peri‐peri, peri‐post, and post‐post). Age at menopause was defined (at baseline and follow‐up) as the last date of menstrual bleeding. The baseline questionnaire collected details on contraceptive (oral or injectable) and menopause hormone therapy (MHT) use.

### Sociodemographic and behavioral factors

The baseline questionnaire captured data on highest attained education level, employment status, and tobacco use. Self‐reported physical activity was quantified using the validated Global Physical Activity Questionnaire (GPAQ), from which minutes per week of moderate to vigorous intensity physical activity (MVPA) were calculated by summing moderate and vigorous work and travel‐related and recreational activities and classified using the physical activity recommendations (≥150 minutes of MVPA per week).^(^ ^34^, ^35^ ^)^

### Anthropometry and muscle strength

Weight (kilograms) was measured to the nearest 0.1 kg using a TANITA digital scale (model: TBF‐410, TANITA Corp., US), while height (meters) was measured to the nearest 0.01 m using a wall‐mounted stadiometer (Holtain, UK). BMI was derived (weight/height^2^: kg/m^2^) and categorized as underweight (<18.5 kg/m^2^), normal weight (18.5–24.99 kg/m^2^), overweight (25.0–29.99 kg/m^2^), or obese (>30.0 kg/m^2^).^(^ ^36^ ^)^ Three nondominant arm grip strength measurements were performed, seated, arm flexed at 90° next to the body, using a Jamar hydraulic handheld dynamometer (JLW Instruments, Chicago, IL, USA) and recording the maximum of three efforts to estimate muscle strength.

### Dual‐energy X‐ray absorptiometry (DXA)

DXA scans were performed twice, at baseline and follow‐up, using the same Hologic Discovery A instrument; scanner performance over the period of the study was monitored using standard manufacturer quality assurance and quality control protocols (Hologic Inc. Bedford, MA, USA; Apex version 13.4.2:3 software; S/N 83145). The scanner was operated by the same trained technician to measure areal BMD of the total body (TB), total hip (TH), and lumbar spine (LS) (L1–L4). The DXA‐derived body composition measurements included total body less head (TBLH) fat and lean tissue mass (as a proxy for muscle mass) in kilograms (excluding the head ensured wigs and hairpieces did not artifactually distort measurements). Coefficients of variation for BMD DXA measurements, repeated three times in 15 participants, were 0.62% and 0.67% for TH and LS regions, respectively. For participants aged ≥50 years, *T*‐scores were calculated using the sex‐specific reference data from the National Health and Nutrition Examination Survey (NHANES) III as recommended by the International Society for Clinical Densitometry (ISCD).^(^ ^37^, ^38^ ^)^

### HIV status

At baseline a voluntary HIV antibody test, Alere Determine™ HIV‐1/2 (Alere San Diego, Inc. San Diego, CA, USA), was offered to all women with a confirmatory second test for those testing HIV positive.^(^ ^39^ ^)^ At follow‐up participants not known to be HIV positive underwent repeat HIV antibody testing (Wondfo One Step HIV–1/2 Whole Blood/Serum/Plasma: Test 2 lines Guanghu Wondfo Biotech Co., Ltd). Participants testing HIV positive were referred to local HIV care services.

### Ethical considerations

Ethical approvals were granted by the Human Research Ethics Committee (Medical) of the University of the Witwatersrand (Certificates M121029, M160604, and M090620). All participants provided written informed consent prior to inclusion in the study, in line with the Declaration of Helsinki.^(^ ^40^ ^)^

### Statistical analysis

Data were analyzed using Stata 15 (StataCorp 2017 Stata Statistical Software, College Station, TX, USA). Consistency checks, for example for HIV status and menopause status, were performed over the two time points. Quantitative variables were assessed for normality and are presented as mean ± SD, or as median and interquartile range (IQR) if not normally distributed. Categorical variables are presented as frequencies and proportions. Comparisons of women living with and without HIV were made using Student's *t* test for normally distributed continuous variables and the Mann‐Whitney U test/K Wallis for non‐normally distributed continuous data. Chi‐squared and Fisher's exact (where numbers were low) tests were used to compare categorical variables.

The primary study outcomes were change in TB, TH, and LS BMD (∆BMD, g/cm^2^), with a positive value indicating a BMD gain and a negative value a BMD loss between baseline and follow‐up. Secondary outcomes were changes in TBLH fat and fat‐free (lean) mass. Correlation coefficients were used to determine linear relationships between exposure variables and ∆BMD in complete case analyses. Univariable linear regression was used to determine associations between baseline characteristics and ∆BMD stratified by baseline HIV status. Multivariable linear regression models, stratified by HIV status, that included a priori *confounders* (age, weight at baseline, ∆weight, follow‐up time) and MT groupings were created to determine whether ∆BMD differed by MT group, with the referent group being those who remained premenopausal throughout. Regression diagnostics were performed to check model assumptions and multicollinearity. Likelihood ratio tests were used to test for level‐specific (i.e., pre‐, peri‐, and postmenopause) interaction between MT groups and HIV status on ∆BMD at the different BMD sites. Sensitivity analyses were performed excluding women who reported (i) a hysterectomy, (ii) use of contraceptives, (iii) use of MHT, and (iv) being HIV negative at baseline and positive at follow‐up to ensure our MT groupings were not biased by their inclusion.

## Results

### Study population

Of 504 women randomly selected to attend follow‐up (from 1004 enrolled at baseline), 450 (89.3%) had DXA scans performed at baseline and follow‐up, confirmed baseline HIV status, and complete menopause stage data (Figure 1). These 450 women were similar to those who were not followed‐up on, had an unknown HIV status, or lacked a DXA scan (*n* = 554), in terms of age, employment, BMI, and physical activity, but were more likely to be pre‐ or perimenopausal and to be living without HIV (Supplementary Table 1). Of the women with LS scans, seven did not have a hip DXA, and 107 (23.8%) did not have a TB DXA (Figure 1).

### Baseline characteristics

Table 1 provides baseline data stratified by HIV status of the participants. Of the 450 women, 191 (42.4%) were postmenopausal at baseline, 293 (65.1%) were obese, and 65 (14.4%) were living with HIV (Table 1). Women with HIV were younger, less likely to have finished high school, and less likely to be obese than women without HIV (Table 1). Unemployment was high (46%). (Time spent in MVPA averaged 4 h/week and 274 [61%] met WHO guidelines of 150 minutes or more MVPA per week), with no differences between those with and without HIV. Mean age increased with advancement in menopause transition group (pre, peri, and post) (Supplementary Table 2). The mean self‐reported age at menopause was 47 (6.2) years and was not different between the two groups. Overall, 38 (8.5%) reported a hysterectomy (15/38 recalled oophorectomy) and 68 (15.8%) reported oral or injectable contraceptive use (Table 1). Women with HIV had less fat (mean difference 4.8 kg [95% confidence interval {CI}: 1.9, 7.8]; *p* = 0.001) and lean mass (mean difference 3.0 kg [95% CI: 0.8, 5.2]; *p* = 0.008) than women without HIV, with marginally lower grip strength (mean difference 1.5 kg [95% CI: −0.02, 2.98]; *p* = 0.053).

**Table 1:** Baseline characteristics among women stratified by HIV status

Indicators	Total (*n* = 450)		HIV + (*n* = 65)		HIV − (*n* = 385)		*p* value
	*N*		*N*		*N*		
**Sociodemographic and behavioral factors**							
Age (years), *median* (*IQR*)	450	49.3 (44.5–54.2)	65	45.0 (43.6–50.7)	385	49.6 (45–54.7)	<0.001
Education, *n* (%)	450		65		385		0.019
Did not finish high school		288 (64.0)		50 (76.9)		238 (61.8)	
Finished high school		162 (36.0)		15 (23.1)		147 (38.2)	
Currently unemployed, *n* (%)	446	205 (46.0)	64	32 (50.0)	382	173 (45.3)	0.484
Ever smoked tobacco, *n* (%)	447	45 (10.1)	64	6 (9.4)	383	39 (10.2)	0.842
Total MVPA (min/week), *n* (%)	450		65		385		0.326
< 150 min/week		176 (39.1)		29 (44.6)		147 (38.2)	
≥ 150 min/week		274 (60.9)		36 (55.4)		238 (61.8)	
**Women's health**							
Menopause stage, *n* (%)	450		65		385		0.533
Premenopause		140 (31.1)		24 (36.9)		116 (30.1)	
Perimenopause		119 (26.4)		15 (23.1)		104 (27.0)	
Postmenopause		191 (42.4)		26 (40.0)		165 (42.9)	
Age at menopause, *median* (*IQR*)	191	47 (44–52)	26	49 (45–50)	165	47 (44–52)	0.0.933
Previous hysterectomy, *n* (%)	446	38 (8.5)	64	2 (3.1)	382	36 (9.4)	0.143
Use of contraceptives (ever), *n* (%)	430	68 (15.8)	59	7 (11.9)	371	61 (16.4)	0.446
Use of MHT (ever), *n* (%)	429	8 (1.9)	61	1 (1.6)	368	7 (1.9)	0.682
**Anthropometry and muscle strength**							
Weight (kg)	450	81.8 (16.1)	65	74.3 (17.8)	385	83.0 (15.5)	<0.001
Height (m)	450	1.58 (0.06)	65	1.57 (0.06)	385	1.59 (0.06)	0.043
BMI, *n* (%)	450		65		385		0.002
Underweight (<18.5 kg/m^2^)		2 (0.4)		1 (1.5)		1 (0.3)	
Normal (18.5–24.9 kg/m^2^)		49 (10.9)		15 (23.1)		34 (8.8)	
Overweight (25.0–29.9 kg/m^2^)		106 (23.6)		16 (24.6)		90 (23.4)	
Obese (≥30.0 kg/m^2^)		293 (65.1)		33 (50.8)		260 (67.5)	
Grip strength (kg), *mean* (*SD*)	339	26.1 (5.15)	54	24.81 (5.07)	285	26.30 (5.14)	0.053
**DXA fat and lean mass**							
TBLH fat mass (kg)	343	33.0 (9.96)	51	28.8 (11.8)	292	33.7 (9.43)	0.001
TBLH lean mass (kg)	343	43.6 (7.46)	51	41.1 (8.54)	292	44.0 (7.18)	0.008
**DXA BMD (g/cm** ^**2**^ **)**							
Lumbar spine (L1‐L4) BMD	450	0.987 (0.145)	65	0.945 (0.143)	385	0.994 (0.144)	0.012
Total hip BMD	443	0.980 (0.143)	62	0.933 (0.140)	381	0.988 (0.143)	0.005
Total body BMD	343	1.089 (0.100)	51	1.089 (0.097)	292	1.089 (0.101)	0.956
**DXA BMD *T*‐score (age ≥ 50 years)**							
Lumbar spine BMD (L1–L4), *n* (%)	205		20		185		0.392
0: ≥ −1		97 (47.3)		7 (35.0)		90 (48.7)	
1: > − 2.5 and < −1		75 (36.6)		8 (40.0)		67 (36.2)	
2: ≤ −2.5		33 (16.1)		5 (25.0)		28 (15.1)	
Total hip BMD, *n* (%)	204		20		184		0.062
0: ≥ −1		156 (76.5)		15 (75.0)		141 (76.6)	
1: > −2.5 and < −1		43 (21.1)		3 (15.0)		40 (21.7)	
2: ≤ −2.5		5 (2.4)		2 (10.0)		3 (1.6)	
Total body BMD, *n* (%)	168		18		150		0.976
0: ≥ −1		101 (60.1)		11 (61.1)		90 (60.0)	
1: > −2.5 and < −1		59 (35.1)		6 (33.3)		53 (35.3)	
2: ≤ −2.5		8 (4.8)		1 (5.6)		7 (4.7)	
*T*‐Score ≤ −2.5 at either the LS or TH	205	34 (13.6)	20	5 (25.0)	185	29 (15.7)	0.288

*Note*: *T*‐score: Generated from non‐Hispanic white reference values (NHANES, 2005); mean (SD) shown for continuous variables unless otherwise indicated.

Abbreviations: BMD, bone mineral density; BMI, body mass index; DXA, dual‐energy X‐ray absorptiometry; HIV, human immunodeficiency virus; IQR, interquartile range; MHT, menopausal hormone therapy; MVPA, moderate‐vigorous physical activity; TBLH, total body less head.

Among the 205 women ≥50 years of age, osteoporosis (BMD *T*‐score ≤ −2.5) most commonly occurred in the LS, with 33 (16.1%) women having a *T*‐score ≤ −2.5; no differences were seen by HIV status. Overall, the prevalence of osteoporosis at either the LS or the TH was 25.0% and 15.7% in women living with and without HIV, respectively (Table 1).

### Menopausal transitions during study period

Women were followed up for a median of 4.8 (IQR: 4.1–5.6) years with no difference in follow‐up time by HIV status (Table 2). In addition, follow‐up time did not differ by menopausal transition status (Supplementary Table 2). Of the 259 women who were either pre‐ or perimenopausal at baseline, 221 (85%) transitioned at least one menopausal stage during the follow‐up period. At follow‐up, 328 (72.7%) were postmenopausal, while only 38 (8.4%) remained premenopausal (Table 2). At follow‐up, of the 81 women with HIV (16 of whom had been HIV negative at baseline), 53 (65.4%) reported taking ART. Medication use data were not available at baseline, but at follow‐up no women reported use of antiresorptive medication.

**Table 2:** Crude changes in anthropometry and BMD through menopausal transition in South African women living with and without HIV

Indicators	Total		HIV + women		HIV − women		*p* value
	*N*		*N*		*N*		
Follow‐up time (years), median (IQR)	450	4.8 (4.1–5.6)	65	4.9 (4.4–5.4)	385	4.9 (4.1–5.6)	0.933
Menopause transition stage, *n* (%)	450		65		385		0.094
Pre‐Pre menopause		38 (8.4)		11 (16.9)		27 (7.0)	
Pre‐Peri menopause		22 (4.9)		3 (4.6)		19 (4.2)	
Pre‐Post menopause		80 (17.8)		10 (15.4)		70 (15.6)	
Peri‐Peri menopause		62 (13.8)		11 (16.9)		51 (13.2)	
Peri‐Post menopause		57 (12.7)		4 (6.2)		53 (13.8)	
Post‐Post menopause		191 (42.2)		26 (40.0)		165 (42.9)	
**∆ Anthropometry and muscle strength**							
Height (cm)	450	−0.313 (1.40)	65	−0.245 (0.97)	385	−0.324 (1.46)	0.673
Weight (kg)	450	0.022 (6.26)	65	1.70 (6.99)	385	−0.26 (6.09)	0.019
BMI (kg/m^2^)	450	0.07 (2.55)	51	0.57 (2.97)	291	−0.02 (2.46)	0.125
Grip strength (kg)	322	1.07 (5.78)	49	2.28 (5.57)	273	0.85 (5.80)	0.112
**∆ DXA fat and lean mass**							
TBLH fat mass (kg)	343	2.07 (4.41)	51	3.23 (5.24)	292	1.87 (4.23)	0.043
TBLH lean mass (kg)	343	−1.72 (4.84)	51	−2.14 (6.13)	292	−1.64 (4.58)	0.503
**∆ DXA BMD (g/cm** ^**2**^ **)**							
Lumbar spine BMD (L1–L4)	450	−0.023 (0.068)	65	−0.032 (0.051)	385	−0.022 (0.071)	0.285
Total hip BMD	443	−0.009 (0.058)	62	−0.009 (0.040)	381	−0.009 (0.060)	0.977
Total body BMD	343	0.001 (0.043)	51	−0.010 (0.047)	292	0.003 (0.042)	0.040
**∆ Bone measurements (*T*‐score) (*n* = 205 for age ≥ 50)**							
Lumbar spine BMD (L1–L4)	205	−0.22 (0.53)	20	−0.28 (0.47)	185	−0.21 (0.53)	0.564
Total hip BMD	202	−0.11 (0.52)	20	−0.03 (0.42)	182	−0.12 (0.53)	0.484
Total body BMD	168	−0.04 (0.51)	18	−0.22 (0.47)	150	−0.02 (0.51)	0.117

*Note*: *T*‐score generated from non‐Hispanic white reference values (NHANES, 2005); mean (SD) shown for continuous variables unless otherwise indicated.

Abbreviations; BMD, bone mineral density; BMI, body mass index; DXA, dual‐energy X‐ray absorptiometry; HIV, human immunodeficiency virus; IQR, interquartile range; TBLH, total body less head.

### Changes in fat and muscle mass

Women with HIV at baseline gained a mean (SD) of 1.7 (7.0) kg in body weight during follow‐up, while women without HIV lost on average 0.26 (6.1) kg; height remained constant. Irrespective of HIV status, fat mass increased, and lean mass decreased, though greater gains in fat mass were seen in women living with HIV at baseline (mean difference in ∆fat mass: 1.36 kg [95% CI: 0.05, 2.67]) (Table 2). Small increases in grip strength were observed, which did not differ by baseline HIV status.

### Changes in BMD

Over the 4.8 years of follow‐up, a reduction in LS BMD (mean: 0.023; SD: 0.068 g/cm^2^) was seen in the overall study population, which corresponded with a mean (SD) reduction in *T*‐score of 0.22 (0.53) in the 205 women ≥50 years of age; however, this was similar between those with and without HIV at baseline (Table 2). Consistent but weak reductions were seen in TH BMD, which did not differ by HIV status. By contrast, while TB BMD was unchanged in women without HIV (mean: 0.003; SD 0.042 g/cm^2^), in the women with HIV at baseline, TB BMD losses were evident (mean: 0.010; SD: 0.047 g/cm^2^).

#### Univariable analyses

Age, education, employment, smoking history, physical activity, fat and lean mass, and grip strength at baseline were not associated with ∆BMD, irrespective of HIV status. However, taller height was inconsistently associated with TB and TH BMD (Table 3).

**Table 3:** Univariable associations between baseline characteristics and change in BMD, stratified by HIV status at follow‐up

	∆ Lumbar spine BMD (g/cm^2^) [95%CI]		∆ Total hip BMD (g/cm^2^) [95% CI]		∆ Total body BMD (g/cm^2^) [95%CI]	
	HIV − (*n* = 385)	HIV + (*n* = 65)	HIV − (*n* = 381)	HIV + (*n* = 62)	HIV − (*n* = 292)	HIV + (*n* = 51)
**Sociodemographic and behavioral factors**						
Age (in decades)	−0.009 [−0.027, 0.010]	−0.009 [−0.036, 0.017]	−0.008 [−0.018, 0.003]	−0.001 [−0.023, 0.021]	−0.009 [−0.018, −0.001]^b^	−0.022 [−0.049, 0.006]
Finished high school	−0.016[−0.038, 0.006]	−0.011[−0.041, 0.019]	0.006[−0.006, 0.019]	−0.008[−0.033, 0.016]	0.003[−0.007, 0.013]	0.004[−0.028, 0.035]
Currently unemployed	−0.001 [−0.023, 0.020]	0.013 [−0.013, 0.038]	−0.008 [−0.021, 0.004]	0.019 [−0.001, 0.040]	0.004 [−0.006, 0.014]	−0.013 [−0.040, 0.014]
Ever smoked tobacco	−0.007 [−0.043, 0.028]	0.015 [−0.028, 0.057]	−0.007 [−0.027, 0.013]	−0.001 [−0.039, 0.037]	0.006 [−0.010, 0.023]	0.003 [−0.042, 0.047]
Meeting MVPA guidelines (≥150 mins/week)	−0.018 [−0.040, 0.004]	−0.017 [−0.042, 0.008]	−0.010 [−0.022, 0.002]	−0.011 [−0.031, 0.010]	−0.004 [−0.014, 0.006]	−0.018 [−0.044, 0.008]
**Women's health**						
Menopause transition stage						
0:Pre‐Pre	Ref	Ref	Ref	Ref	Ref	Ref
1:Pre‐Peri	−0.032 [−0.093, 0.030]	−0.079 [−0.134, −0.023]^b^	−0.033 [−0.067, 0.001]	−0.028 [−0.081, 0.025]	−0.025 [−0.055, 0.004]	−0.068 [−0.122, −0.015]^b^
2:Pre‐Post	−0.049 [−0.095, −0.003]^b^	−0.069 [−0.105, −0.032]^a^	−0.052 [−0.078, −0.026]^a^	−0.030 [−0.065, 0.006]	−0.031 [−0.054, −0.009]^b^	−0.064 [−0.104, −0.024]^a^
3:Peri‐Peri	0.033 [−0.016, 0.082]	−0.009 [−0.045, 0.027]	−0.002 [−0.029, 0.026]	−0.001 [−0.036, 0.034]	−0.003 [−0.027, 0.021]	0.002 [−0.039, 0.043]
4:Peri‐Post	−0.037 [−0.085, 0.012]	−0.094 [−0.143, −0.045]^a^	−0.038 [−0.065, −0.010]^a^	−0.015 [−0.062, 0.033]	−0.041 [−0.064, −0.018]^a^	−0.080 [−0.134, −0.027]^b^
5:Post‐Post	−0.034 [−0.077, 0.008]	−0.058 [−0.088, −0.027]^a^	−0.025 [−0.049, −0.001]^b^	−0.025 [−0.055, 0.006]	−0.032 [−0.053, −0.011]^a^	−0.053 [−0.086, −0.020]^b^
**Anthropometry and muscle strength**						
Weight (10 kg)	0.0008 [−0.0059, 0.0077]	0.0041 [−0.0031, 0.0112]	0.0015 [−0.0024, 0.0054]	−0.0004 [−0.0062, 0.0055]	0.0008 [−0.0024, 0.0040]	0.0017 [−0.0057, 0.0092]
Height (m)	−0.038 [−0.221, 0.145]	0.136 [−0.075, 0.347]	−0.056 [−0.159, 0.048]	0.195 [0.027, 0.362]^b^	−0.091 [−0.178, −0.003]^b^	0.182 [−0.069, 0.433]
Grip strength (kg)	−0.001 [−0.004, 0.002]	0.001 [−0.002, 0.003]	0.001 [−0.0002, 0.003]	0.001 [−0.001, 0.003]	0.0004 [−0.001, 0.002]	−0.001 [−0.003, 0.002]
**DXA fat and lean mass**						
TBLH fat mass (kg)	0.0001 [−0.001, 0.002]	0.0003 [−0.001, 0.002]	0.0004 [−0.0003, 0.001]	−0.0003 [−0.001, 0.001]	−0.00001 [−0.001, 0.001]	0.0001 [−0.001, 0.001]
TBLH lean mass (kg)	−0.001 [−0.003, 0.001]	0.0002 [−0.002, 0.002]	−0.0004 [−0.001, 0.001]	−0.0001 [−0.001, 0.001]	0.001 [−0.0001, 0.001]	0.001 [−0.001, 0.002]

*Note*: Univariable linear regression generating beta coefficients [95% CI] indicating absolute change in BMD (g/cm^2^) according to baseline characteristics.

Abbreviations: BMD, bone mineral density; HIV, human immunodeficiency virus; MVPA, moderate to vigorous physical activity; TBLH, total body less head.

^a^ *p* < 0.001.

^b^ *p* < 0.05.

**Fig. 2:**
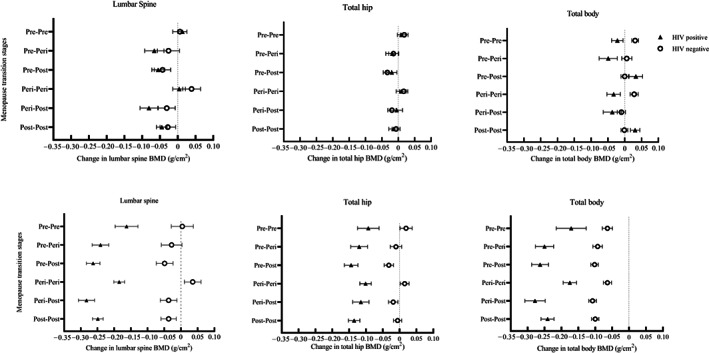
Absolute changes in BMD in menopause transition stages among women living with and without HIV (top row: unadjusted) (bottom row: adjusted for age and weight at baseline, change in weight, and follow‐up time). Graphs show univariable and multivariable effects of menopause transition on LS, TH, and TB BMD in top and bottom rows, respectively. Linear regression estimates are shown; *β* (standard error) representing g/cm^2^ losses/gains in BMD over follow‐up period as distinguished by a reference line (*x* = 0). Individuals with HIV are indicated by triangles and those without HIV by circles. The prevalence of HIV in this population was 14.4%, and at follow‐up, most women (72.9%) were postmenopausal. Evidence of interaction between pre‐post, post‐post MT groups and HIV status on TB BMD was detected in adjusted analyses.

When analyzing women in the six MT groups, changes in LS, TH, and TB BMD were observed in women both with and without HIV (Figure 2; top row). Compared to the 38 (8.4%) women who remained premenopausal, most MT groups showed losses in LS and TB BMD and, to a lesser extent, TH BMD, with losses greatest in those who transitioned through to postmenopause (e.g., pre‐post and peri‐post MT groups) or who were postmenopausal throughout (Table 3). In women with HIV, LS and TB BMD losses in some of the groups were approximately double those seen in women without HIV infection. Furthermore, in women with HIV, transitioning from premenopause to perimenopause was associated with the greatest losses in LS and TB BMD, a pattern not seen in women without HIV (Table 3).

#### Multivariable analyses

After adjustment for a priori *confounders* (age, follow‐up time, baseline weight, and ∆weight), absolute differences in **∆**BMD at all three skeletal sites were clearly seen, such that women with HIV showed evidence of substantially greater bone losses than women without HIV, in all MT groups (Figure 2; bottom row). Compared to women who remained premenopausal (reference group), all women who transitioned from pre‐ to postmenopause showed significant declines in BMD at all three skeletal sites, whether or not they had HIV (Table 4). Being postmenopausal at follow‐up was associated with BMD losses, particularly at TB and TH in women without HIV, and particularly the TB and LS in women with HIV (Table 4).

**Table 4:** Relative change in BMD in women with and without HIV according to menopause transition stage, compared to women who remained premenopausal, after adjusting for age, weight, change in weight, and follow‐up time

	∆ Lumbar spine BMD (g/cm^2^) [95%CI]		∆ Total hip BMD (g/cm^2^) [95%CI]		∆ Total body BMD (g/cm^2^) [95%CI]	
	HIV − (*n* = 385)	HIV + (*n* = 65)	HIV − (*n* = 381)	HIV + (*n* = 62)	HIV − (*n* = 292)	HIV + (*n* = 51)
Follow‐up time (years)	−0.010 [−0.022, 0.003]	−0.004 [−0.016, 0.009]	−0.007 [−0.014, −0.0002]^b^	0.003 [−0.009, 0.016]	0.016 [0.010, 0.022]^a^	0.014 [−0.004, 0.033]
Age at baseline (decade)	0.010 [−0.009, 0.033]	0.036 [0.010, 0.061]^b^	0.003 [−0.001, 0.014]	0.019 [−0.007, 0.046]	0.002 [−0.007, 0.011]	0.026 [−0.006, 0.058]
Menopause transition stage						
0:Pre‐Pre	Ref	Ref	Ref	Ref	Ref	Ref
1:Pre‐Peri	−0.032 [−0.093, 0.029]	−0.078 [−0.129, −0.028]^b^	−0.030 [−0.064, 0.004]	−0.027 [−0.078, 0.023]	−0.029 [−0.057, −0.001]^b^	−0.079 [−0.133, −0.025]^b^
2:Pre‐Post	−0.053 [−0.101, −0.004]^b^	−0.100 [−0.144, −0.064]^a^	−0.052 [−0.078, −0.025]^a^	−0.051 [−0.091, −0.011]^b^	−0.038 [−0.060, −0.016]^a^	−0.092 [−0.142, −0.042]^a,i^
3:Peri‐ Peri	0.031 [−0.017, 0.080]	−0.022 [−0.055, 0.011]	−0.004 [−0.030, 0.023]	−0.008 [−0.041, 0.025]	0.0001 [−0.023, 0.023]	−0.004 [−0.045, 0.037]
4:Peri‐Post	−0.041 [−0.091, 0.009]	−0.120 [−0.167, −0.067]^a^	−0.038 [−0.065, −0.010]^b^	−0.021 [−0.069, 0.026]	−0.044 [−0.067, −0.023]^a^	−0.107 [−0.167, −0.047]^a^
5:Post‐Post	−0.041 [−0.086, 0.005]	−0.085 [−0.118, −0.054]^a^	−0.025 [−0.051, −0.0002]^b^	−0.042 [−0.076, −0.009]^b^	−0.036 [−0.057, −0.015]^a^	−0.070 [−0.108, −0.031]^a,i^
Weight at baseline (10 kg)	0.0008 [−0.0076, 0.0061]	0.0055 [−0.0001, 0.0112]	0.0023 [−0.0015, 0.0060]	0.0003 [−0.0053, 0.0059]	0.0007 [−0.0023, 0.0037]	0.0027 [−0.0039, 0.0092]
∆Weight (10 kg)	−0.0008 [−0.0166, 0.0151]	0.0181 [0.0040, 0.0322]	0.0019 [0.0102, 0.0276]^a^	0.0222 [0.0081, 0.0362]^b^	0.0001 [−0.0069, 0.0067]	−0.0009 [−0.0167, 0.001]

*Note*: Multivariable linear regression generating beta coefficients [95% CI] indicating the absolute change in BMD (g/cm^2^). Model included age, baseline weight, ∆weight, follow‐up time, and six menopausal transition groups with comparison to pre‐pre menopause transition group (^a^ *p* < 0.001, ^b^ *p* < 0.05, ^i^ *p* < 0.05 for interaction of menopause transition by HIV status).

Abbreviations: BMD, bone mineral density; HIV, human immunodeficiency virus.

Compared to women who remained premenopausal, the effect of transitioning from pre‐ to postmenopause was associated with greater TB BMD loss in women with HIV (−0.092 g/cm^2^ [−0.042, −0.142]; *p* = 0.001), than in women without HIV (−0.038 g/cm^2^ [−0.016, −0.060]; *p* = 0.001; HIV*pre‐post menopause interaction *p* = 0.034). Similarly, postmenopausal women (post‐post) with HIV lost more TB BMD (−0.070 g/cm^2^ [−0.031, −0.108], *p* = 0.001) than women without HIV (−0.036 g/cm^2^ [−0.015, −0.057], *p* = 0.001, HIV*post‐post menopause interaction *p* = 0.049) (Table 4). In addition, postmenopausal women living with HIV lost a greater amount of TH BMD (−0.042 g/cm^2^ [−0.076, −0.009], *p* = 0.015) compared to women living without HIV (−0.025 g/cm^2^ [−0.051, −0.0002], *p* = 0.051). Compared to women who remained premenopausal, the effect of transitioning from pre‐ to postmenopause was associated with LS BMD losses in both women with HIV (−0.100 g/cm^2^ [−0.144, −0.064], *p* < 0.001) and without HIV (−0.053 g/cm^2^ [−0.004, −0.101], *p* = 0.033, HIV*pre‐post menopause interaction *p* = 0.705). Although postmenopausal women (post‐post) with HIV lost LS BMD (−0.085 g/cm^2^ [−0.054, −0.118], *p* < 0.001), losses were modest in women without HIV (−0.041 g/cm^2^ [0.005, −0.086], *p* = 0.078, HIV*post‐post menopause interaction *p* = 0.551) (Table 4). Reassuringly, women who remained perimenopausal showed no evidence of bone loss. In women with and without HIV, weight gain was associated with gains in TH, but not LS or TB BMD, independent of age, MT, baseline weight, and follow‐up time (Table 4).

In sensitivity analyses excluding women reporting (i) hysterectomy +/−oophorectomy, (ii) contraceptive use, and (iii) MHT use, results were similar and conclusions unchanged. At follow‐up, 16 women who had tested negative at baseline tested positive for HIV infection (by follow‐up, 13 had initiated ART); excluding these 16 women from the analyses did not change the overall conclusions (Supplementary Tables 3–7).

## Discussion

We have shown that urban‐dwelling South African women lose bone following menopause and that these bone losses, across all skeletal sites, are greater in those living with HIV compared with women without HIV, despite an increase in body weight over this period. Taken together, our findings show that living with HIV at the time of menopause is associated with detrimental body composition changes, more specifically a decrease in BMD and an increase in fat mass, with potential implications for both future fracture and metabolic risks.

We have shown that, as in other populations globally,^(^ ^41^ ^)^ black South African women transitioning through menopause experience substantial declines in BMD across all skeletal sites, and those transitioning into or who are in postmenopause exhibit the greatest losses. Our data add to the findings of a small cross‐sectional study of 68 black South African women that showed that postmenopausal women had lower LS and hip BMD than pre‐ or perimenopausal women^(^ ^42^ ^)^ and earlier results from 709 black South African women in Soweto, from which this follow‐up cohort was derived, which found lower TB BMD in postmenopausal than pre‐ or perimenopausal women.^(^ ^43^ ^)^ Our data are novel in their longitudinal nature in SSA.

Notably, ours is the first study to investigate the interaction between HIV and menopause on bone health in a southern African population. Importantly, we have shown that black South African women living with HIV who transition through to postmenopause lose bone particularly at the TB and LS. Our results differ when compared to a smaller 2‐year longitudinal study of 120 South African postmenopausal women living with HIV and established on ART, which showed little change in TB or LS BMD, potentially due to the small sample size and short follow‐up period.^(^ ^44^ ^)^ Our evidence for HIV infection augmenting the effect of postmenopausal estrogen loss on bone concurs with a recent, albeit small, longitudinal study of 128 postmenopausal Hispanic and African American women in North America that showed greater DXA‐measured bone loss at the LS and forearm in those with HIV, though a formal interaction was not explored.^(^ ^20^ ^)^ Notably, findings from North Americans with African ancestry cannot be generalized to black South African populations.^(^ ^45^ ^)^ The mechanism underlying the interaction between HIV infection and menopause is unclear, although the loss of anabolic estrogen, together with the chronic inflammatory effects of HIV, would be expected to promote bone resorption and net bone loss. The multiethnic SWAN study (US) reported that LS BMD declined by 0.015–0.03 g/cm^2^ per year in the late perimenopausal and postmenopausal periods, equating to losses of 0.06 to 0.12 g/cm^2^ over 4 years of follow‐up.^(^ ^46^ ^)^ Consistent with this, we identified LS BMD declines of 0.085 g/cm^2^ in postmenopausal women living with HIV, which equate to a 9.0% LS BMD loss over the study period. Furthermore, the TH BMD declines of 0.042 g/cm^2^ in postmenopausal women living with HIV equate to 4.5% TH BMD declines over the follow‐up period. These losses are associated with approximately 50%–100% (1.5 to 2 times) higher fracture rates, based on a previous meta‐analysis.^(^ ^47^ ^)^

In our study cohort, 81.5% of women with HIV were receiving ART. Although we lacked data on ART regimes, at the time of this study the recommended first‐line regime for adults in South Africa was tenofovir disopoxil fumerate (TDF), lamivudine (3TC), and efavirenz (EFV) as a fixed‐dose combination.^(^ ^48^ ^)^ TDF is specifically known to cause 1%–3% greater BMD loss in the first 2 years of therapy^(^ ^49^, ^50^ ^)^ by impairing renal tubular function to cause hypophosphaturia and reduced skeletal mineralization.^(^ ^8^ ^)^ This demineralization could partially explain the losses particularly seen in TB BMD, which reflects 80% cortical bone and is the body's mineral reservoir, and the trabecular‐rich LS. Trabecular bone has a large surface area for mineral exchange and is more metabolically active than cortical bone, rapidly mobilizing mineral when needed.^(^ ^51^ ^)^ Furthermore, EFV has been associated with low BMD at the hip in young (mean age 35 years) South African men and women.^(^ ^52^ ^)^

In our study, osteoporosis was relatively common in the women aged ≥50 years, affecting 13.6% overall, and 25% of those with HIV. As the number of women aged ≥50 years with osteoporosis and HIV were few (*n* = 5), we were underpowered to detect a difference by HIV status. The HIV prevalence observed among our reported urban‐dwelling women was slightly lower than that recently observed among rural‐dwelling women with HIV in South Africa, of whom 37% had a *T*‐score ≤ −2.5.^(^ ^53^ ^)^ Use of MHT was rare, despite strong evidence that it can help prevent bone fragility.^(^ ^54^ ^)^ However, in South Africa, MHT is often accessed through private healthcare services, which may explain the low use in this less‐affluent community in Soweto.^(^ ^55^ ^)^

Most participants were classified as having obesity, despite the majority (61%) meeting WHO recommendations of 150 minutes or more of MVPA per week.^(^ ^35^ ^)^ During the 5‐year follow‐up, the women studied gained fat mass, regardless of HIV status, although women living with HIV experienced the greater gains. Until recently it had been thought that fat mass was protective against fracture. Although a study of black South African postmenopausal women reported an inverse association between fat mass and fracture risk score (rather than fracture incidence),^(^ ^56^ ^)^ fat may negatively affect bone through several pathways, including by altering bone‐regulating hormones, increased oxidative stress and inflammation, and altered bone cell metabolism reducing bone quality.^(^ ^57^ ^)^ A 25‐year longitudinal study in Finnish postmenopausal women reported that those with obesity were 30% more likely to experience hip fractures at a younger age than normal‐weight women, with higher postfracture mortality.^(^ ^58^ ^)^ A meta‐analysis of 25 prospective cohort studies showed that upper‐arm fractures were more common in women with high BMI, and when adjusted for BMD, women with a BMI ≥35 kg/m^2^ were 16% more likely to experience a fragility fracture compared with normal‐weight women.^(^ ^59^ ^)^ Furthermore, the Global Longitudinal study of Osteoporosis in Women (GLOW) has demonstrated increased ankle and upper‐leg fractures in those with obesity.^(^ ^59^ ^)^ The reasons for the site‐specific effect of fat mass on fracture risk may reflect fat mass distributions, excessive loading, how bone sites function to resist fracture depending on the predominant failure mechanism at that site, and where you land from a fall.^(^ ^60^, ^61^ ^)^ The WHO predicts that, by 2050, the population of older South African women will grow by 15.4%,^(^ ^62^ ^)^ and, combined with the increasing prevalence of overweight and obesity,^(^ ^63^ ^)^ the implications for bone health and fracture incidence are of concern.

### Strength and limitations

This is the first longitudinal study of middle‐aged women to investigate menopause‐associated bone loss in a population with a high HIV prevalence in Africa. Limitations include lack of data at baseline on an ART regimen, CD4 counts and viral loads, parity, and breastfeeding, all of which are associated with bone mass.^(^ ^50^, ^64^, ^65^, ^66^, ^67^ ^)^ We lacked complete data on femoral neck BMD, which prevented inclusion in analyses and limits clinical relevance as this site is particularly associated with hip fragility fractures. Low numbers of women with HIV in each of the MT groups limited power to detect differences by HIV status. We performed complete case analyses for TB and TH adjustments as fewer women had data on these sites when compared to the LS. In the absence of population‐specific reference data, we used NHANES III reference data to calculate *T*‐scores for women age ≥ 50 years, though these are not yet validated in an African population,^(^ ^37^ ^)^ and artifactual increases in LS BMD due to degenerative disease cannot be excluded. The relatively young mean age at FMP is likely due to poor recall in women 3 or more years post‐FMP, as was reported previously^(^ ^33^ ^)^; however, this would not be expected to cause misclassification in our analyses. We lacked data on fracture incidence, which would have provided additional insights. Given increased HIV‐related morbidity and mortality, the study may have underestimated the effect of HIV on the skeleton, as those with severe disease may have been unable to participate.^(^ ^68^ ^)^

## Conclusions

In urban South Africa, women transitioning through menopause lose bone, and these losses are exacerbated by the coexistence of HIV infection. Given the high prevalence of HIV in southern Africa and increasing longevity, these findings raise a concern over future fracture burdens in the region. Menopause‐associated bone loss is highly treatable, yet few women in this study reported using such treatments, raising concerns over equitable access to healthcare. The HIV clinics in South Africa could consider routine bone health assessment in older menopausal women, and new tools (i.e., the new South African FRAX tool)^(^ ^69^ ^)^ are now available for this purpose. Future longitudinal studies on fracture incidence in aging women with HIV are warranted.

## Author contributions

**Tafadzwa Madanhire:** Conceptualization; data curation; formal analysis; methodology; visualization; writing – original draft. **Julia H. Goedecke:** Writing – review and editing. **Kate A. Ward:** Writing – review and editing. **Nicole Jaff:** Writing – review and editing. **Nigel J. Crowther:** Writing – review and editing. **Shane Norris:** Writing – review and editing. **Rashida A. Ferrand:** Writing – review and editing. **Andrea M. Rehman:** Writing – review and editing. **Lisa K. Micklesfield:** Conceptualization; methodology; supervision; visualization; writing – original draft; writing – review and editing. **Celia L. Gregson:** Conceptualization; formal analysis; methodology; supervision; visualization; writing – original draft; writing – review and editing.

## Disclosure

The authors declare that no competing interests exist.

### Peer review

The peer review history for this article is available at https://publons.com/publon/10.1002/jbmr.4765.

## Supporting information


**Data S1.** Supporting Information

## Data Availability

The data that support the findings of this study are available on request from the senior authors. The data are not publicly available due to privacy/ethical restrictions.
