# Supplementary material for: The Impact of Human Immunodeficiency Virus and Menopause on Bone Mineral Density: A Longitudinal Study of Urban‐Dwelling South African Women
Source: J Bone Miner Res. 2023 Feb 1;38(5):619–30. doi: 10.1002/jbmr.4765 (PMC10946789; doi:10.1002/jbmr.4765)
Supplement: Supplementary file 1 — Data S1. Supporting Information [file JBMR-38-619-s001.docx]

**Supplementary materials**

**The impact of HIV on bone mineral density through menopause: a longitudinal study of urban dwelling South African women.** Madanhire et al

**Participants’ characteristics at baseline**

Supplementary table 1: Baseline characteristics of study participants according to whether they did or did not have follow-up data available

| **Indicators** | **Total**  **(n=1004)** | | **Follow-up**  **(n=450)** | | **No follow-up**  **(n=554)** | | **p-value** |
| --- | --- | --- | --- | --- | --- | --- | --- |
|  | **N** |  | **N** |  | **N** |  |  |
| **Socio-demographic & behavioral factors** | | | | | | | |
| Age (years), *median (IQR)* | 1004 | 49.4 (44.7-54) | 450 | 49.3 (44.5-54.2) | 554 | 49.5 (45-53.9) | 0.911 |
| Education, n (%)  Did not finish high school  Finished high school | 860 | 565 (65.7)  295 (34.3) | 450 | 288 (64.0)  162 (36.0) | 410 | 277 (67.6)  133 (32.4) | 0.272 |
| Currently unemployed, n (%) | 1000 | 455 (45.2) | 446 | 205 (46.0) | 554 | 250 (44.6) | 0.676 |
| Ever smoked tobacco, n (%) | 1001 | 106 (10.5) | 447 | 45 (10.1) | 554 | 61(10.8) | 0.721 |
| Total MVPA (minutes/week), *median (IQR)* | 1004 | 210 (59.9-900) | 450 | 240 (60-990) | 554 | 190 (59.9-840) | 0.223 |
| **Women’s health** | | | | | | | |
| Menopausal stage, n (%)  Pre-menopause  Peri-menopause  Post-menopause | 879 | 261(29.7)  208 (23.7)  410 (46.6) | 450 | 140 (31.1)  119 (26.4)  191(42.4) | 429 | 121(28.2)  89 (20.8)  219 (51.1) | 0.028 |
| Use of MRT (ever), n (%) | 958 | 15 (1.6) | 429 | 8 (1.9) | 529 | 7 (1.3) | 0.502 |
| **Clinical assessments and medical history** | | | | | | | |
| HIV status, n (%)  Infected  Uninfected | 699 | 118 (16.9)  581 (83.1) | 450 | 65 (14.4)  385 (85.6) | 249 | 53 (21.3)  196 (78.7) | 0.021 |
| **Anthropometry & muscle strength** | | | | | | | |
| Weight (kg), *median (IQR)* | 823 | 81.8 (16.5) | 450 | 81.9 (16.2) | 373 | 81.6 (16.9) | 0.756 |
| Height (metres) | 1004 | 1.580 (0.06) | 450 | 1.584 (0.058) | 554 | 1.577 (0.056) | 0.045 |
| BMI, n (%)  Underweight (<18.5)  Normal (18.5-24.9)  Overweight (25.0-29.9)  Obese (≥30.0) | 821 | 6 (0.7)  88 (10.7)  177 (21.6)  550 (67.0) | 450 | 2 (0.4)  45 (10.0)  104 (23.1)  299 (66.4) | 371 | 4 (1.1)  43 (11.6)  73 (19.7)  251 (67.7) | 0.428 |

*BMI: Body Mass Index; BMD: Bone Mineral Density; HRT: Hormone Replacement Therapy; MVPA: Moderate-Vigorous Physical Activity, mean (SD) shown for continuous variables unless otherwise indicated*

**Age at baseline and follow-up time**

Supplementary table 2: Follow-up times and age distribution for menopausal transition and menopausal groups respectively

| **Indicators** | **Total** | | **HIV + women** | | **HIV - women** | | **p value** |
| --- | --- | --- | --- | --- | --- | --- | --- |
|  | **N** |  | **N** |  | **N** |  |  |
| Age at baseline (years),  Pre-menopause  Peri-menopause  Post-menopause | 140  119  191 | 46.9 (43.1-48.1)  48.4 (47.4-51.5)  52.4 (48.5-56.2) | 24  15  26 | 44.1 (41.5-47.4)  46.4 (43.2-49.0)  49.0 (45.6-52.6) | 116  104  165 | 47.2 (43.7-49.4)  49.3 (45.9-51.6)  53.0 (48.6-56.6) | 0.063  0.403  0.001 |
| Follow up time (years), median (IQR) | 450 | 4.8 (4.1-5.6) | 65 | 4.9 (4.4-5.4) | 385 | 4.9 (4.1-5.6) | 0.933 |
| Follow-up times for menopause transition (years), median (IQR)  Pre- Pre menopause  Pre-Peri menopause  Pre-Post menopause  Peri- Peri menopause  Peri-Post menopause  Post- Post menopause | 38  22  80  62  57  191 | 4.6 (4.0-5.4)  4.9 (3.9-5.6)  5.1 (4.2-5.7)  4.8 (3.8-5.3)  4.9 (4.1-5.5)  4.9 (4.3-5.6) | 11  3  10  11  4  26 | 4.8 (3.8-5.5)  5.3 (4.7-5.4)  5.0 (4.6-5.5)  4.7 (4.0-5.0)  4.8 (4.1-5.4)  4.8 (4.4-5.2) | 27  19  70  51  53  165 | 4.5 (3.9-5.2)  4.9 (3.8-5.6)  5.1 (4.1-5.5)  4.9 (4.1-5.3)  4.9 (4.1-5.5)  4.9 (4.3-5.6) | 0.520  0.416  0.839  0.832  0.731  0.613 |

**Sensitivity analysis for adjusted changes in BMD, in women with and without HIV, according to menopause transition, age, weight, change in weight and follow-up time.**

Supplementary table 3: Sensitivity analysis for the multivariate model excluding participants with a hysterectomy/+- oophorectomy (n=38)

|  | ∆ **Lumbar Spine BMD (g/cm^2)^) [95%CI]** | | ∆ **Total Hip BMD (g/cm^2)^) [95%CI]** | | ∆ **Total Body BMD (g/cm^2)^) [95%CI]** | |
| --- | --- | --- | --- | --- | --- | --- |
|  | **HIV – (n=346)** | **HIV + (n=62)** | **HIV – (n=343)** | **HIV + (n=60)** | **HIV – (n=263)** | **HIV + (n=49)** |
| Follow-up time (years) | -0.011 [-0.025, 0.003] | -0.004 [-0.017, 0.009] | -0.005 [-0.012, 0.002]^b^ | 0.004 [-0.009, 0.017] | 0.016 [0.009, 0.022]^a^ | 0.018 [0.0003, 0.035]^b^ |
| Age at baseline (decade) | 0.013 [-0.011, 0.036] | 0.035 [0.009, 0.062]^b^ | 0.001 [-0.010, 0.012] | 0.018 [-0.009, 0.046] | 0.002 [-0.008, 0.012] | 0.024 [-0.006, 0.055] |
| Meno- transition stages  0:Pre- Pre  1:Pre-Peri  2:Pre-Post  3:Peri- Peri  4:Peri-Post  5:Post-Post | Ref  -0.033 [-0.103, 0.037]  -0.051 [-0.105, 0.002]  0.033 [-0.021, 0.086]  -0.042 [-0.096, 0.013]  -0.041 [-0.091, 0.010] | Ref  -0.083 [-0.144, 0.021]^b^  -0.100 [-0.142, -0.058]^a^  -0.023 [-0.058, 0.013]  -0.120 [-0.170, -0.070]^a^  -0.086 [-0.121, -0.052]^a^ | Ref  -0.023 [-0.057, 0.011]  -0.049 [-0.075, -0.023]^a^  -0.003 [-0.029, 0.023]  -0.034 [-0.061, -0.007]^b^  -0.024 [-0.048, 0.001] | Ref  -0.016 [-0.077, 0.045]  -0.050 [-0.092, -0.008]^b^  -0.007 [-0.043, 0.028]  -0.021 [-0.070, 0.029]  -0.042 [-0.077, -0.006]^b^ | Ref  -0.025 [-0.056, 0.006]  -0.036 [-0.060, -0.013]^a^  0.002 [-0.022, 0.026]  -0.042 [-0.067, -0.019]^a^  -0.031 [-0.053, -0.009]^a^ | Ref  -0.047 [-0.108, 0.014]  -0.095 [-0.144, -0.046]^ai^  -0.008 [-0.049, 0.033]  -0.110 [-0.168, -0.051]^a^  -0.073 [-0.111, -0.034]^ai^ |
| Weight at baseline (10 kg) | -0.0010 [-0.0086, 0.0066] | 0.0055 [-0.0002, 0.0111] | 0.0010 [-0.0027, 0.0047] | 0.0003 [-0.0054, 0.0059] | 0.0013 [-0.0019, 0.0045] | 0.0023 [-0.0040, 0.0085] |
| ∆Weight ( 10 kg) | -0.0005 [-0.0166, 0.0175] | 0.0185 [0.0042, 0.0329]^b^ | 0.0204 [0.0121, 0.0288]^a^ | 0.0222 [0.0080, 0.0363]^a^ | 0.0009 [-0.0061, 0.0080] | 0.0012 [-0.0161, 0.0138] |

*Multivariable linear regression generating beta coefficients [95% CI] indicating the absolute change in BMD (in g/cm^2^). Model included age, ∆weight, weight, follow-up time and 6 menopausal transition groups with comparison to the pre-pre menopause transition group ( ^a^p<0.001, ^b^p<0.05 ^i^p-value for interaction of menopause transition by HIV status <0.05)*

Supplementary table 4: Sensitivity analysis for the multivariate model excluding participants on menopause replacement therapy (n=8)

|  | ∆ **Lumbar Spine BMD (g/cm^2)^) [95%CI]** | | ∆ **Total Hip BMD (g/cm^2)^) [95%CI]** | | ∆ **Total Body BMD (g/cm^2)^) [95%CI]** | |
| --- | --- | --- | --- | --- | --- | --- |
|  | **HIV – (n=378)** | **HIV + (n=64)** | **HIV – (n=374)** | **HIV + (n=61)** | **HIV – (n=289)** | **HIV + (n=50)** |
| Follow-up time (years) | -0.011 [-0.023, 0.002] | -0.004 [-0.017, 0.009] | -0.008 [-0.015, -0.001]^b^ | 0.003 [-0.010, 0.016] | 0.016 [0.010, 0.022]^a^ | 0.016 [-0.002, 0.034] |
| Age at baseline (decade) | 0.012 [-0.010, 0.033] | 0.036 [0.010, 0.063]^b^ | 0.002 [-0.010, 0.014] | 0.022 [-0.005, 0.050] | 0.0003 [-0.009, 0.010] | 0.022 [-0.010, 0.054] |
| Meno- transition stages  0:Pre- Pre  1:Pre-Peri  2:Pre-Post  3:Peri- Peri  4:Peri-Post  5:Post-Post | Ref  -0.029 [-0.092, 0.033]  -0.048 [-0.098, 0.002]  0.032 [-0.018, 0.082]  -0.039 [-0.090, 0.012]  -0.039 [-0.085, 0.008] | Ref  -0.079 [-0.130, 0.028]^b^  -0.099 [-0.140, -0.059]^a^  -0.022 [-0.056, 0.011]  -0.121 [-0.169, -0.072]^a^  -0.086 [-0.119, -0.054]^a^ | Ref  -0.028 [-0.062, 0.006]  -0.049 [-0.076, -0.021]^a^  -0.004 [-0.032, 0.023]  -0.036 [-0.064, -0.008]^b^  -0.022 [-0.048, 0.004] | Ref  -0.028 [-0.079, 0.023]  -0.049 [-0.090, -0.009]^b^  -0.009 [-0.042, 0.025]  -0.024 [-0.072, 0.025]  -0.044 [-0.078, -0.010]^b^ | Ref  -0.024 [-0.053, 0.004]  -0.030 [-0.053, -0.008]^a^  0.004 [-0.019, 0.028]  -0.039 [-0.062, -0.016]^a^  -0.030 [-0.051, -0.009]^a^ | Ref  -0.077 [-0.131, -0.023]^a^  -0.096 [-0.146, -0.046]^ai^  -0.003 [-0.044, 0.037]  -0.102 [-0.162, -0.043]^a^  -0.068 [-0.106, -0.030]^ai^ |
| Weight at baseline (10 kg) | -0.0017 [-0.0087, 0.0053] | 0.0056 [-0.0001, 0.0112] | 0.0015 [-0.0023, 0.0054] | 0.0004 [-0.0052, 0.0061] | 0.00001 [-0.0029, 0.0031] | 0.0024 [-0.0041, 0.0090] |
| ∆Weight (10 kg) | -0.0008 [-0.0168, 0.0151] | 0.002 [0.0031, 0.0323]^b^ | 0.0187 [0.0099, 0.0274]^a^ | 0.0208 [0.0062, 0.0353]^a^ | -0.0004 [-0.0068, 0.0067] | 0.0014 [-0.0146, 0.0176] |

*Multivariable linear regression generating beta coefficients [95% CI] indicating the absolute change in BMD (in g/cm^2^). Model included age, ∆weight, weight, follow-up time and 6 menopausal transition groups with comparison to the pre-pre menopause transition group ( ^a^p<0.001, ^b^p<0.05 ^i^p-value for interaction of menopause transition by HIV status <0.05)*

Supplementary table 5: Sensitivity analysis for the multivariate model excluding participants on contraceptives (n=68)

|  | ∆ **Lumbar Spine BMD (g/cm^2)^) [95%CI]** | | ∆ **Total Hip BMD (g/cm^2)^) [95%CI]** | | ∆ **Total Body BMD (g/cm^2)^) [95%CI]** | |
| --- | --- | --- | --- | --- | --- | --- |
|  | **HIV – (n=324)** | **HIV + (n=58)** | **HIV – (n=320)** | **HIV + (n=55)** | **HIV – (n=243)** | **HIV + (n=44)** |
| Follow-up time (years) | -0.011 [-0.026, 0.002] | -0.001 [-0.016, 0.013] | -0.007 [-0.015, 0.0004] | 0.007 [-0.007, 0.021] | 0.017 [0.011, 0.023]^a^ | 0.019 [-0.001, 0.039] |
| Age at baseline (decade) | 0.011 [-0.014, 0.036] | 0.038 [0.009, 0.066]^b^ | 0.004 [-0.009, 0.018] | 0.015 [-0.013, 0.043] | 0.001 [-0.009, 0.012] | 0.022 [-0.010, 0.054] |
| Meno- transition stages  0:Pre- Pre  1:Pre-Peri  2:Pre-Post  3:Peri- Peri  4:Peri-Post  5:Post-Post | Ref  -0.039 [-0.110, 0.033]  -0.053 [-0.108, 0.002]  0.045 [-0.011, 0.100]  -0.034 [-0.091, 0.023]  -0.042 [-0.093, 0.010] | Ref  -0.082 [-0.136, 0.028]^a^  -0.102 [-0.149, -0.054]^a^  -0.022 [-0.057, 0.013]  -0.105 [-0.159, -0.051]^a^  -0.086 [-0.122, -0.050]^a^ | Ref  -0.036 [-0.074, 0.002]  -0.052 [-0.081, -0.023]^a^  -0.0004 [-0.029, 0.030]  -0.032 [-0.062, -0.002]^b^  -0.022 [-0.050, 0.004] | Ref  -0.028 [-0.079, 0.024]  -0.049 [-0.096, -0.003]^b^  -0.008 [-0.042, 0.027]  -0.018 [-0.070, 0.034]  -0.036 [-0.072, -0.003]^b^ | Ref  -0.025 [-0.057, 0.007]  -0.035 [-0.059, -0.011]^a^  0.005 [-0.020, 0.031]  -0.041 [-0.065, -0.016]^a^  -0.034 [-0.057, -0.011]^a^ | Ref  -0.080 [-0.138, -0.022]^a^  -0.079 [-0.143, -0.015]^a^  -0.003 [-0.047, 0.041]  -0.086 [-0.155, -0.016]^b^  -0.063 [-0.106, -0.019]^a^ |
| Weight at baseline (10 kg) | 0.0004 [-0.0078, 0.0086] | 0.0047 [-0.0015, 0.0109] | 0.0028 [-0.0015, 0.0072] | 0.0011 [-0.0049, 0.0070] | 0.0010 [-0.0024, 0.0044] | 0.0008 [-0.0065, 0.0081] |
| ∆Weight (10 kg) | -0.0002 [-0.0018, 0.0181] | 0.0151 [-0.0005, 0.0307] | 0.0204 [0.0108, 0.0300]^a^ | 0.0240 [0.0091, 0.0389]^a^ | 0.0030 [-0.0043, 0.0103] | 0.0028 [-0.0020, 0.0144] |

*Multivariable linear regression generating beta coefficients [95% CI] indicating the absolute change in BMD (in g/cm^2^). Model included age, ∆weight, weight, follow-up time and 6 menopausal transition groups with comparison to the pre-pre menopause transition group ( ^a^p<0.001, ^b^p<0.05 ^i^p-value for interaction of menopause transition by HIV status <0.05)*

Supplementary table 6: Sensitivity analysis for the multivariate model excluding participants on contraceptives (n=68) and menopause replacement therapy (n=8)

|  | ∆ **Lumbar Spine BMD (g/cm^2)^) [95%CI]** | | ∆ **Total Hip BMD (g/cm^2)^) [95%CI]** | | ∆ **Total Body BMD (g/cm^2)^) [95%CI]** | |
| --- | --- | --- | --- | --- | --- | --- |
|  | **HIV – (n=318)** | **HIV + (n=57)** | **HIV – (n=314)** | **HIV + (n=54)** | **HIV – (n=241)** | **HIV + (n=43)** |
| Follow-up time (years) | -0.013 [-0.027, 0.002] | -0.002 [-0.016, 0.013] | -0.008 [-0.016, -0.0005]^b^ | 0.007 [-0.007, 0.021] | 0.016 [0.010, 0.022]^a^ | 0.020 [0.0003, 0.040]^b^ |
| Age at baseline (decade) | 0.011 [-0.015, 0.036] | 0.039 [0.009, 0.068]^b^ | 0.004 [-0.010, 0.017] | 0.017 [-0.012, 0.047] | 0.0004 [-0.010, 0.011] | 0.018 [-0.019, 0.054] |
| Meno- transition stages  0:Pre- Pre  1:Pre-Peri  2:Pre-Post  3:Peri- Peri  4:Peri-Post  5:Post-Post | Ref  -0.038 [-0.110, 0.034]  -0.050 [-0.106, 0.006]  0.043 [-0.012, 0.100]  -0.033 [-0.091, 0.024]  -0.042 [-0.094, 0.010] | Ref  -0.083 [-0.137, -0.028]^b^  -0.100 [-0.149, -0.052]^a^  -0.023 [-0.058, 0.013]  -0.105 [-0.160, -0.051]^a^  -0.087 [-0.123, -0.051]^a^ | Ref  -0.036 [-0.074, 0.002]  -0.050 [-0.079, -0.021]^a^  -0.002 [-0.032, 0.027]  -0.031 [-0.061, -0.001]^b^  -0.020 [-0.047, -0.007] | Ref  -0.029 [-0.078, 0.023]  -0.047 [-0.094, -0.0002]  -0.008 [-0.043, 0.027]  -0.020 [-0.072, 0.033]  -0.038 [-0.075, -0.001]^b^ | Ref  -0.025 [-0.056, 0.007]  -0.032 [-0.056, -0.007]^b^  0.005 [-0.020, 0.030]  -0.040 [-0.065, -0.016]^a^  -0.033 [-0.056, -0.011]^a^ | Ref  -0.079 [-0.136, -0.021]^a^  -0.088 [-0.154, -0.023]^a^  -0.003 [-0.046, 0.041]  -0.083 [-0.152, -0.014]^b^  -0.061 [-0.104, -0.018]^a^ |
| Weight at baseline (10 kg) | 0.0006 [-0.0090, 0.0078] | 0.0048 [-0.0015, 0.0111] | 0.0020 [-0.0024, 0.0064] | 0.0012 [-0.0048, 0.0072] | 0.0006 [-0.0028, 0.0039] | 0.0004 [-0.0069, 0.0077] |
| ∆Weight (10 kg) | -0.0003 [-0.0185, 0.0180] | 0.0143 [-0.0021, 0.0307] | 0.0199 [0.0103, 0.0295]^a^ | 0.0225 [0.0068, 0.0382]^a^ | 0.0028 [-0.0045, 0.0100] | -0.0002 [-0.0177, 0.0182] |

*Multivariable linear regression generating beta coefficients [95% CI] indicating the absolute change in BMD (in g/cm^2^). Model included age, ∆weight, weight, follow-up time and 6 menopausal transition groups with comparison to the pre-pre menopause transition group ( ^a^p<0.001, ^b^p<0.05 ^i^p-value for interaction of menopause transition by HIV status <0.05)*

Supplementary table 7: Sensitivity analysis for the multivariate model excluding participants who changed their HIV status (n=16)

|  | ∆ **Lumbar Spine BMD (g/cm^2)^) [95%CI]** | | ∆ **Total Hip BMD (g/cm^2)^) [95%CI]** | | ∆ **Total Body BMD (g/cm^2)^) [95%CI]** | |
| --- | --- | --- | --- | --- | --- | --- |
|  | **HIV – (n=369)** | **HIV + (n=65)** | **HIV – (n=365)** | **HIV + (n=62)** | **HIV – (n=282)** | **HIV + (n=51)** |
| Follow-up time (years) | -0.009 [-0.022, 0.003] | -0.004 [-0.016, 0.009] | -0.005 [-0.012, -0.002] | 0.003 [-0.009, 0.016] | 0.017 [0.011, 0.023]^a^ | 0.014 [-0.004, 0.033] |
| Age at baseline (decade) | 0.009 [-0.013, 0.032] | 0.036 [0.010, 0.061]^a^ | 0.0002 [-0.012, 0.012] | 0.019 [-0.007, 0.046] | 0.001 [-0.008, 0.011] | 0.026 [-0.006, 0.058] |
| Meno- transition stages  0:Pre- Pre  1:Pre-Peri  2:Pre-Post  3:Peri- Peri  4:Peri-Post  5:Post-Post | Ref  -0.025 [-0.088, 0.039]  -0.049 [-0.098, 0.001]  0.029 [-0.021, 0.079]  -0.033 [-0.086, 0.019]  -0.037 [-0.084, 0.010] | Ref  -0.078 [-0.129, -0.028]^b^  -0.100 [-0.144, -0.064]^a^  -0.022 [-0.055, 0.011]  -0.120 [-0.167, -0.067]^a^  -0.086 [-0.118, -0.054]^a^ | Ref  -0.024 [-0.059, 0.010]  -0.051 [-0.078, -0.024]^a^  -0.003 [-0.030, 0.024]  -0.033 [-0.062, -0.005]^b^  -0.022 [-0.048, -0.003] | Ref  -0.027 [-0.078, 0.023]  -0.051 [-0.091, -0.011]^b^  -0.008 [-0.041, 0.025]  -0.022 [-0.069, 0.026]  -0.042 [-0.076, -0.009]^b^ | Ref  -0.030 [-0.057, -0.001]^b^  -0.038 [-0.060, -0.015]^a^  0.0001 [-0.023, 0.023]  -0.042 [-0.065, -0.019]^a^  -0.035 [-0.056, -0.014]^a^ | Ref  -0.079 [-0.133, -0.025]^b^  -0.092 [-0.142, -0.042]^ai^  -0.004 [-0.045, 0.037]  -0.107 [-0.167, -0.047]^a^  -0.070 [-0.108, -0.031]^ai^ |
| Weight at baseline (10 kg) | -0.0021 [-0.0092, 0.0051] | 0.0055 [-0.0001, 0.0112] | 0.0017 [-0.0021, 0.0056] | 0.0003 [-0.0053, 0.0060] | 0.0006 [-0.0025, 0.0036] | 0.0027 [-0.0039, 0.0092] |
| ∆Weight (10 kg) | -0.0011 [-0.0176, 0.0155] | 0.0181 [0.0040, 0.0322] | 0.0161 [0.0071, 0.0250]^a^ | 0.0222 [0.0081, 0.0362]^a^ | 0.0008 [-0.0062, 0.0077] | -0.0009 [-0.0167, 0.0147] |

*Multivariable linear regression generating beta coefficients [95% CI] indicating the absolute change in BMD (in g/cm^2^). Model included age, ∆weight, weight, follow-up time and 6 menopausal transition groups with comparison to the pre-pre menopause transition group ( ^a^p<0.001, ^b^p<0.05 ^i^p-value for interaction of menopause transition by HIV status <0.05)*
